# Dimeric 2G12 as a Potent Protection against HIV-1

**DOI:** 10.1371/journal.ppat.1001225

**Published:** 2010-12-16

**Authors:** Xin M. Luo, Margarida Y. Y. Lei, Rana A. Feidi, Anthony P. West, Alejandro Benjamin Balazs, Pamela J. Bjorkman, Lili Yang, David Baltimore

**Affiliations:** Division of Biology, California Institute of Technology, Pasadena, California, United States of America; Harvard University, United States of America

## Abstract

We previously showed that broadly neutralizing anti-HIV-1 antibody 2G12 (human IgG1) naturally forms dimers that are more potent than monomeric 2G12 in in vitro neutralization of various strains of HIV-1. In this study, we have investigated the protective effects of monomeric versus dimeric 2G12 against HIV-1 infection in vivo using a humanized mouse model. Our results showed that passively transferred, purified 2G12 dimer is more potent than 2G12 monomer at preventing CD4 T cell loss and suppressing the increase of viral load following HIV-1 infection of humanized mice. Using humanized mice bearing IgG “backpack” tumors that provided 2G12 antibodies continuously, we found that a sustained dimer concentration of 5–25 µg/ml during the course of infection provides effective protection against HIV-1. Importantly, 2G12 dimer at this concentration does not favor mutations of the HIV-1 envelope that would cause the virus to completely escape 2G12 neutralization. We have therefore identified dimeric 2G12 as a potent prophylactic reagent against HIV-1 in vivo, which could be used as part of an antibody cocktail to prevent HIV-1 infection.

## Author Summary

Most successful vaccines function by eliciting antibodies that bind to the surface of pathogens of interest from the host immunologic repertoire. This should also be the case for an HIV-1 vaccine, but broadly neutralizing anti-HIV-1 antibodies have proven hard to elicit with any reagent. Thus, methods to directly administer broadly neutralizing anti-HIV-1 antibodies, such as passive transfusion, become appealing. It is therefore important to find out which antibodies, or antibody cocktails, would provide effective protection against HIV-1 before administering them. Here, we show that the dimeric fraction of 2G12, a unique monoclonal anti-HIV-1 antibody that dimerizes naturally, provides better protection against HIV-1 than its monomeric fraction. As an added bonus, although HIV-1 can evolve to completely escape antibody control, the 2G12 dimer does not favor such evolution. Our study suggests that the 2G12 dimer may be a suitable reagent for direct administration to protect people from HIV-1 infection.

## Introduction

Human efficacy trials of vaccine candidates designed to elicit antibody-based immunity against HIV-1 have mostly failed [1], [2], raising questions as to whether such an approach to HIV-1 vaccination is at all feasible. A recent human vaccine trial in Thailand [3], however, provided a promising signal of efficacy. While there is no direct evidence of which component of the vaccine was effective, it could be antibody-based immunity. In the trial, 98.6% of vaccinated individuals produced “binding antibodies” against HIV-1 envelope protein gp120 although no broadly neutralizing antibodies. The possibility that antibody-mediated protection was effective has reenergized the search for effective anti-HIV-1 antibodies.

Existing broadly neutralizing anti-HIV-1 antibodies are valuable starting points for generating protection against HIV-1. Several broadly neutralizing antibodies have been proposed as the basis for designing protective mechanisms against HIV-1 in recent years [4], [5]. Among them, 2G12 is unique, because it recognizes a constellation of carbohydrates on gp120 [6], [7], [8], [9] and has an unusual structure that involves a domain swap between the two heavy chains [8]. 2G12 is most effective at neutralizing clade B strains of HIV-1 [10].

A series of studies have described the in vivo protective effects of 2G12 against simian/HIV-1 in macaques [11], [12], [13] and against HIV-1 in humans [14], [15], [16], [17]. Interestingly, in the studies where 2G12 was combined with other broadly neutralizing antibodies such as 4E10 and 2F5 [16], [17], 2G12 provided the dominant protective effect against HIV-1. The relatively long in vivo half-life of 2G12 can partially explain this phenomenon [18]. However, albeit protective, 2G12 also selected HIV-1 escape mutants in vivo [16], [19]; therefore, it is important to identify a new reagent or method to minimize the rate of appearance of such escape mutants.

We have previously shown that 2G12 IgG1 can form natural dimers that are 50-80–fold more potent than monomeric 2G12 IgG1 in in vitro neutralization of various strains of HIV-1 [20]. 2G12 monomer, in common with typical IgGs, contains two antigen-binding Fabs and one Fc region, but the heavy chain regions of the Fabs are domain-swapped to create a single (Fab)_2_ unit [8]. 2G12 dimer contains four Fabs and two Fcs, which form a structure, presumably through inter-molecular domain swapping, that does not interconvert with 2G12 monomer [20]. The present study was designed to investigate the in vivo potency of dimeric 2G12 in controlling HIV-1 infection in a humanized mouse model. We show that dimeric 2G12 is effective at providing protection against HIV-1 without selecting viral mutants that would completely escape 2G12 neutralization, suggesting that the 2G12 dimer is a suitable prophylactic reagent for use against HIV-1.

## Results

### Dimeric 2G12 is more potent than monomeric 2G12 in vivo after passive transfer

The dimeric form of the monoclonal antibody 2G12 possesses increased in vitro neutralization potency compared to the monomeric form [20]. It is unknown, however, whether dimeric 2G12 would have a long enough half-life to be more effective than the 2G12 monomer at preventing HIV-1 infection in vivo. To address this question, we prepared separate stocks of purified 2G12 monomer and dimer and passively transferred 0.5 mg/mouse of 2G12 monomer or dimer into Rag2^−/−^γ_c_^−/−^ mice reconstituted with human immune cells (Supporting Figure S1A). We then challenged the mice intravenously (i.v.) with the CCR5-tropic strain of HIV-1, JR-CSF, at a dose of 400 ng of p24.

**Figure 1:**
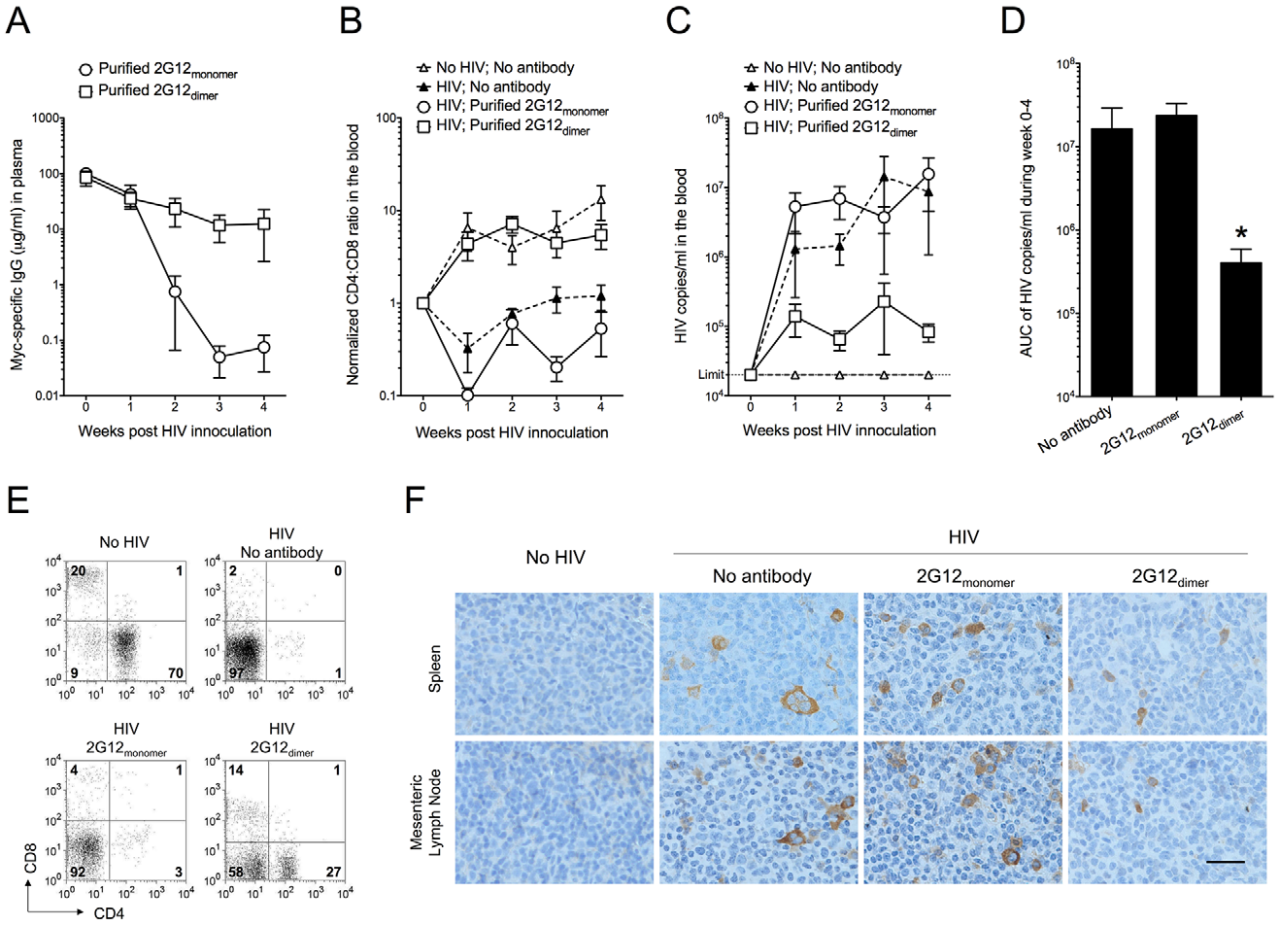
Protection against HIV-1 infection in humanized mice by passively transferred 2G12 dimer. Humanized mice were injected (i.v.) with 0.5 mg of purified 2G12 monomer (n = 6) or 2G12 dimer (n = 5) at 4 months of age and challenged by HIV-1_JR-CSF_ (i.v.; 400 ng of p24) one day after passive transfer. (A) Concentrations of 2G12 in the mouse plasma. 2G12 was measured by Myc-specific ELISA and data are shown as average ± s.e.m. All data shown from now on are average ± s.e.m. unless a bar graph is shown, where the data are presented as average + s.e.m. (B) The ratios of human CD4:CD8 in the peripheral blood. CD4 and CD8 cell populations were measured weekly by flow cytometry. The ratios of CD4:CD8, after normalization to week-0 values (set as 1), were plotted. Mice with no antibody but HIV-1 challenge (n = 7) and mice with neither antibody nor HIV-1 (n = 6) were used as controls. The CD4:CD8 ratio in this humanized mouse model increased with the age of the mice as seen in the “No HIV; No antibody” control. Therefore, all mice involved in this study were age-matched. One-way analysis of variance (ANOVA) with Tukey's multiple comparison posttest showed that the group with 2G12 dimer was not different from the No HIV-1 control (*p*>0.05); however, it had significantly higher CD4:CD8 ratios than the group with 2G12 monomer (*p*<0.05). All data points in each treatment group, regardless of time, were included in the ANOVA. (C) Viral load in the mouse plasma. Viral RNA was extracted from mouse plasma and the viral load was measured. The detection limit of the assay was 20,000 HIV-1 copies/ml of mouse plasma. The differences were not statistically significant. (D) Total viral load from week 0 to week 4. Area under the curve (AUC) of the viral load during week 0-4 was calculated and plotted. The group with 2G12 dimer had significantly lower viral load than the other two groups based on individual Mann-Whitney tests (*p* = 0.0303 between 2G12 dimer and No antibody; *p* = 0.0159 between the 2G12 dimer and 2G12 monomer). (E) Percentages of human CD4 and CD8 T cells in the spleen after 4 weeks. Plots shown were pre-gated on CD3 T cells. (F) Immunohistochemical analysis of HIV-1 p24 in the spleen and mesenteric lymph node after 4 weeks. The images were taken using an Olympus BX51 microscope with 400× magnification (Scale bar, 25 µm).

Using an ELISA targeting a Myc tag fused to the light chain of the purified antibodies, we found that the concentration of 2G12 monomer declined quickly in the mouse plasma whereas the 2G12 dimer was relatively stable (Figure 1A). The elimination (β phase) half-lives of the purified human IgGs in the humanized mouse plasma were estimated as 3.5±0.9 days for the 2G12 dimer and 0.9±0.2 days for the 2G12 monomer. The 2G12 dimer prevented CD4 T cell loss in the peripheral blood following HIV-1 infection, whereas the 2G12 monomer did not provide protection (Figure 1B). In addition, the 2G12 dimer moderately suppressed the increase of viral load in the blood (Figure 1C), causing an overall reduction of 97.5% in viral load compared to the control that lacked antibody (Figure 1D). The 2G12 monomer, on the other hand, did not suppress the increase of viral load following HIV-1 infection (Figure 1C). We also analyzed the percentages of T cells and the numbers of p24^+^ cells in the spleen, thymus, and mesenteric lymph node. As shown in Figure 1E, we found that without 2G12, HIV-1 almost completely depleted CD4^+^ cells in the spleen. The percentage of splenic CD8^+^ cells also decreased, presumably because they rely on CD4^+^ T helper cells for proliferation and survival [21]. Between the two forms of 2G12, the monomer had a minimal effect at preventing the loss of CD4^+^ and CD8^+^ splenocytes following HIV-1 infection, whereas the 2G12 dimer was able to rescue nearly half of the CD4^+^ cells and most of CD8^+^ cells in the spleen (Figure 1E). A similar effect was observed in the mesenteric lymph node (Figure S1B) but not in the thymus (Figure S1C), presumably because a CCR5-tropic virus was used and there are few CCR5^+^ T cells in the thymus [22]. Immunohistochemical analysis using an antibody against HIV-1 p24 confirmed that the 2G12 dimer was effective at limiting HIV-1 infection in both the spleen and the mesenteric lymph node (Figure 1F). HIV-1 p24^+^ cells were hardly found in the thymus (data not shown).

These results demonstrated increased protection against HIV-1 of purified 2G12 dimer compared to 2G12 monomer when the antibodies were administered to humanized mice prior to HIV-1 challenge.

### 2G12 dimer provided continuously at a low level is sufficient for protection against HIV-1

**Figure 2:**
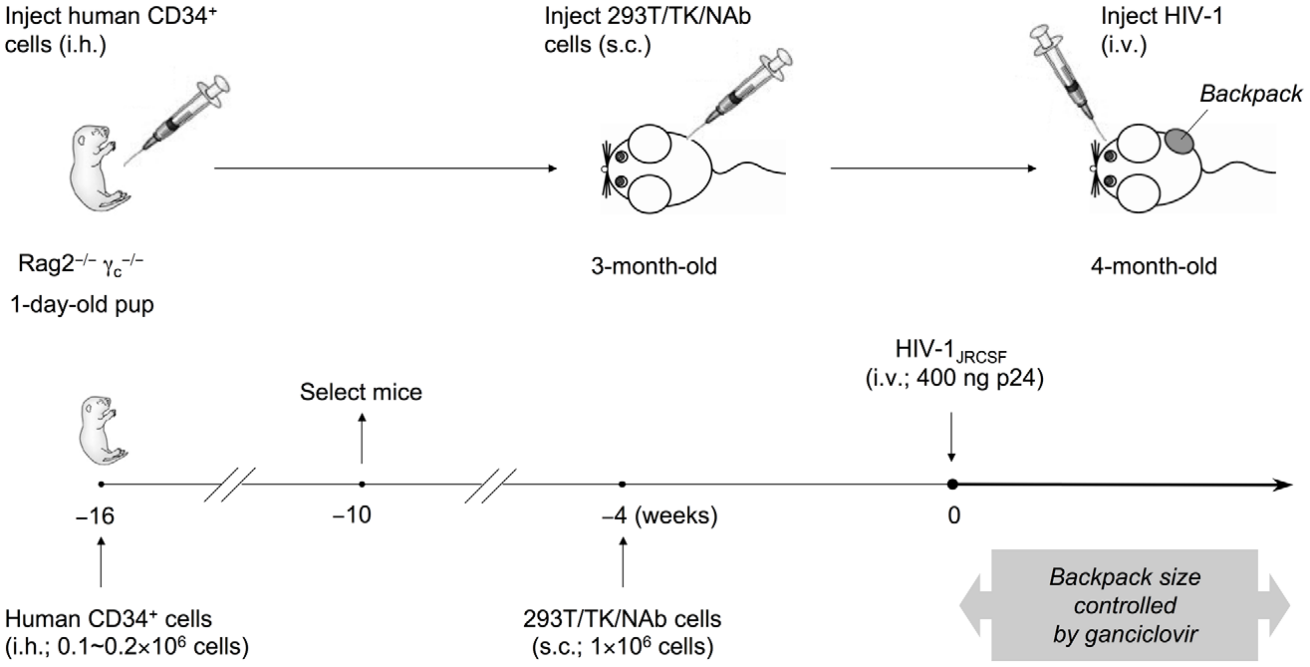
Generation of humanized mice with IgG “backpack” tumors. Rag2^−/−^γ_c_^−/−^ mice were intrahepatically (i.h.) injected with 0.1-0.2×10^6^ human CD34^+^ hematopoietic stem and progenitor cells at 1 day of age. The humanized mice were screened for CD45^+^ human cells at 6 weeks of age, and mice with good reconstitution were selected for the following procedures. First, to achieve a sustained level of anti-HIV-1 neutralizing antibodies (NAb) in mice, we delivered the antibodies through subcutaneous (s.c.) injection of a cell line on the right side of the lower back when the mice were 3-months-old. The cell line, 293T/TK/NAb, formed controllable packs on the lower back of the mice. The backpack size was closely monitored biweekly and the drug ganciclovir was injected (i.p.) after HIV-1 challenge and when the backpacks exceeded the size limit of 1.5 cm^2^. The concentrations of 2G12 produced in the blood were monitored by ELISA. Second, to establish HIV-1 infection in these mice, the JR-CSF strain of the virus was injected intravenously (i.v.) at a dose of 400 ng p24 when the backpacked mice were approximately 4-month-old. The infected mice were monitored weekly for the percentages of T cell populations, the HIV-1 viral load, and the concentrations of 2G12 in the blood. They were sacrificed 4 weeks after HIV-1 inoculation and the blood and tissues were analyzed.

To investigate whether the higher potency of 2G12 dimer compared to the monomer resulted only from its longer in vivo half-life, we modified the conventional humanized mice [23], [24] to carry antibody-expressing cells as backpacks [25] that produced antibodies continuously throughout the course of HIV-1 infection (Figure 2). This strategy avoided the dramatic fluctuation of antibody concentrations that usually occur when antibodies were administered through multiple administrations [16], [17]. The antibody-expressing cells were injected subcutaneously (s.c.) and formed localized backpacks whose size could be controlled by the administration of ganciclovir, a prodrug that killed backpacked cells co-expressing herpes simplex virus thymidine kinase (TK) along with the antibody [26]. Because the backpack size positively correlated with the concentration of 2G12 in the blood (Figure S2A), we could control the backpack size to limit the antibody concentration within a reasonably small range.

We made mice with backpacks that expressed wild-type 2G12 (named “2G12 BP”) and those with backpacks expressing D2, a mutant of 2G12 that is expressed with an increased dimer/monomer ratio [20] (named “D2 BP”). We previously reported that wild-type 2G12 cells produce 78% monomer and 22% dimer whereas the D2 clone produced 60% monomer and 40% dimer; and that the monomers and dimers produced by wild-type 2G12 or D2 2G12 exhibited no significant differences in biophysical and neutralization characteristics [20]. Since the 2G12 monomer and dimer share the same heavy and light chains, an ELISA would not distinguish between the two forms, making it difficult to directly measure the dimer:monomer ratios in the backpacked mice. Size exclusion chromatography, which could be normally used to determine relative levels of monomer and dimer, would require several milliliters of mouse blood for each sample collection, which was not feasible. Instead, we calculated the monomer:dimer ratios based on the production ratios of monomer versus dimer in the two cell lines (3.5: 1 for 2G12 BP and 1.5: 1 for D2 BP) and their individual half-lives in the humanized mice (see Materials and Methods for details). We then used the ratios to estimate the concentrations of 2G12 dimer and 2G12 monomer in the blood samples (Table 1; concentrations of total 2G12 and 2G12 dimer are shown; the concentration of 2G12 monomer can be obtained by subtracting the dimer concentration from the concentration of total 2G12).

**Table 1:** Concentrations of total 2G12 and 2G12 dimer (average ± s.e.m.) in backpacked mice.

	2G12 BP (µg/ml)						D2 BP (µg/ml)						BP 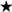 (µg/ml)					
Weeks after HIV-1 injection	Total			Dimer			Total			Dimer			Total			Dimer		
0	1.7	±	0.9	0.9	±	0.5	7.1	±	3.2^b^	5.1	±	2.4^d^	110.4	±	14.9	58.2	±	7.9
1	3.2	±	1.4	1.7	±	0.7	8.6	±	2.2^b^	6.2	±	1.6^d^	224.8	±	46.6	118.5	±	24.6
2	5.7	±	1.9	3.0	±	1.0	12.9	±	2.5	9.3	±	1.8^d^	448.9	±	105.0	236.7	±	55.4
3	7.2	±	2.3	3.8	±	1.2	17.7	±	2.3^b^	12.8	±	1.6^d^	293.0	±	88.0	154.4	±	46.4
4	31.5	±	4.9	16.6	±	2.6	34.4	±	1.6	24.8	±	1.2	208.3	±	21.5	109.8	±	11.4
AUC^a^	37.1	±	6.8	17.2	±	3.8	61.3	±	6.0^c^	44.2	±	4.3^e^	1139.0	±	158.2	506.1	±	87.0

Plasma concentrations of 2G12 (Total) were measured by ELISA. 2G12 dimer and 2G12 monomer concentrations were then calculated as described in the text and Materials and Methods. Concentrations of total 2G12 and 2G12 dimer are shown; the concentration of 2G12 monomer for each condition is obtained by subtracting the dimer concentration from the concentration of total 2G12. BP, backpacks. BP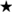, large backpacks that yield high concentrations of 2G12. Ganciclovir was injected to control the size of the backpacks below 1.5 cm^2^.

a AUC: area under the curve from week 0 to week 4.

b Significant difference in total 2G12 concentration between D2 BP and 2G12 BP. Mann-Whitney tests showed that the *p* values for week 0, 1, 3 were 0.0256, 0.0228, 0.0221, respectively.

c Significant difference in AUC of total 2G12 between D2 BP and 2G12 BP (Mann-Whitney test; *p* = 0.0411).

d Significant difference in 2G12 dimer concentration between D2 BP and 2G12 BP. Mann-Whitney tests showed that the *p* values for week 0, 1, 2, 3 were 0.0198, 0.0084, 0.0197, 0.014, respectively.

e Significant difference in AUC of 2G12 dimer between D2 BP and 2G12 BP (Mann-Whitney test; *p* = 0.0047).

**Figure 3:**
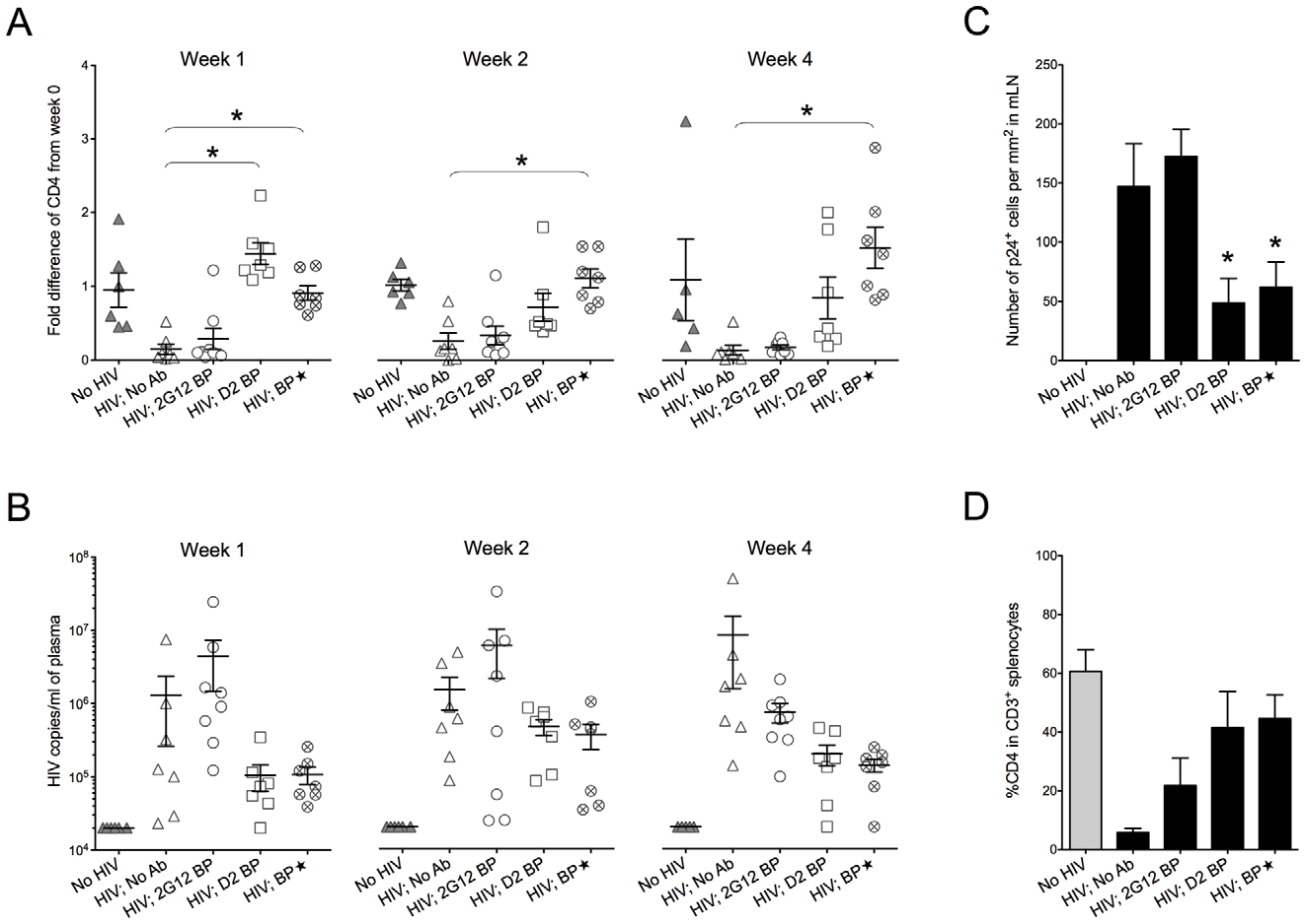
Protection against HIV-1 infection in humanized mice by a sustained low level of dimeric 2G12. Backpacked mice were generated as described in Figure 2. Backpacks expressing wild-type 2G12 were named “2G12 BP” (n = 8) whereas the ones expressing D2 mutant were named “D2 BP” (n = 7). Another group of mice (n = 7) were made to carry large wild-type 2G12 backpacks (“BP
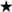
”) that generated >100 µg/ml of 2G12 monomer plus dimer in the blood before HIV-1 inoculation. The concentrations of total 2G12 and 2G12 dimer are shown in Table 1. (A) Fold differences of %CD4 T cells in the peripheral blood from week 0 to week 1, week 2, or week 4. CD4 T cells were measured weekly by flow cytometry. One-way ANOVA with Tukey's multiple comparison posttest showed that the group with D2 BP had a significantly higher percentage of CD4 T cells than the “HIV-1; No Ab” control (n = 7) at week 1 (*p*<0.05). The BP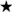 group had significantly higher percentages of CD4 T cells than the “HIV-1; No Ab” group for week 1, week 2, and week 4 (*p*<0.05). (B) Viral load in the mouse plasma. The detection limit of the assay was 20,000 HIV-1 copies/ml of mouse plasma. The differences were not statistically significant. The virus was not detectable in two different mice in the D2 BP group at week 1 and week 4, respectively. One mouse in the BP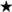 group also had undetectable viral load at week 4. The D2 BP mice had an averaged 90% reduction in viral load from the “HIV; No Ab” control at week 1 and 4. At week 2, the averaged reduction was 70%. (C) Number of p24^+^ cells in the mesenteric lymph node (mLN). Four weeks after HIV-1 challenge, mLNs were harvested, fixed, and sectioned for immunohistochemical analysis of HIV-1 p24. The numbers of p24^+^ cells were counted manually and presented as the number of cells per mm^2^ area of the specimen. One-way ANOVA with Tukey's multiple comparison posttest showed that both D2 BP and BP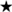 groups had significantly lower numbers of p24^+^ cells in mLN than the HIV-1 groups with 2G12 BP or no antibody (*p*<0.05). (D) Percentage of CD4 T cells in CD3^+^ splenocytes. Mice were sacrificed after 4 weeks and human T cells in their spleens were analyzed by flow cytometry. The groups were not significantly different.

The D2 BP provided an estimated 3-5-fold more dimer than the 2G12 BP during the first 3 weeks of HIV-1 infection (Table 1; *p*<0.02 for weeks 0, 1, 2 and 3). The concentrations of 2G12 monomer were not significantly different between the D2 BP and 2G12 BP groups at each time point (*p*>0.05) although the combined 2G12 concentrations were higher in the D2 BP group due to significantly greater dimer concentrations. Analysis of the peripheral blood lymphocytes showed that 2G12 BP barely had any protective effect on CD4 T cells compared to the control that lacked antibody (Figure 3A, weeks 1, 2 and 4). In contrast, D2 BP effectively protected CD4 T cells from being cleared by HIV-1 after one week of infection (Figure 3A, week 1; *p*<0.05). D2 BP also appeared to offer some protection for CD4 T cells 2 and 4 weeks after HIV-1 inoculation although the effect was not statistically significant. Analysis of HIV-1 copy numbers in the mouse plasma showed that D2 BP moderately suppressed the viral load at each time point (Figure 3B) and significantly suppressed the overall viral load (Figure S2B; *p*<0.01), suggesting that D2 BP is potent at preventing viral entry and/or eliminating HIV-1 from the circulation. The mice with D2 BP also had significantly lower numbers of p24^+^ cells in the mesenteric lymph node than mice carrying 2G12 BP (Figure 3C), although neither backpack significantly protected the spleen from HIV-1 infection (Figure 3D for the percentage of CD4 T cells and Figure S2C for the number of p24^+^ cells).

**Figure 4:**
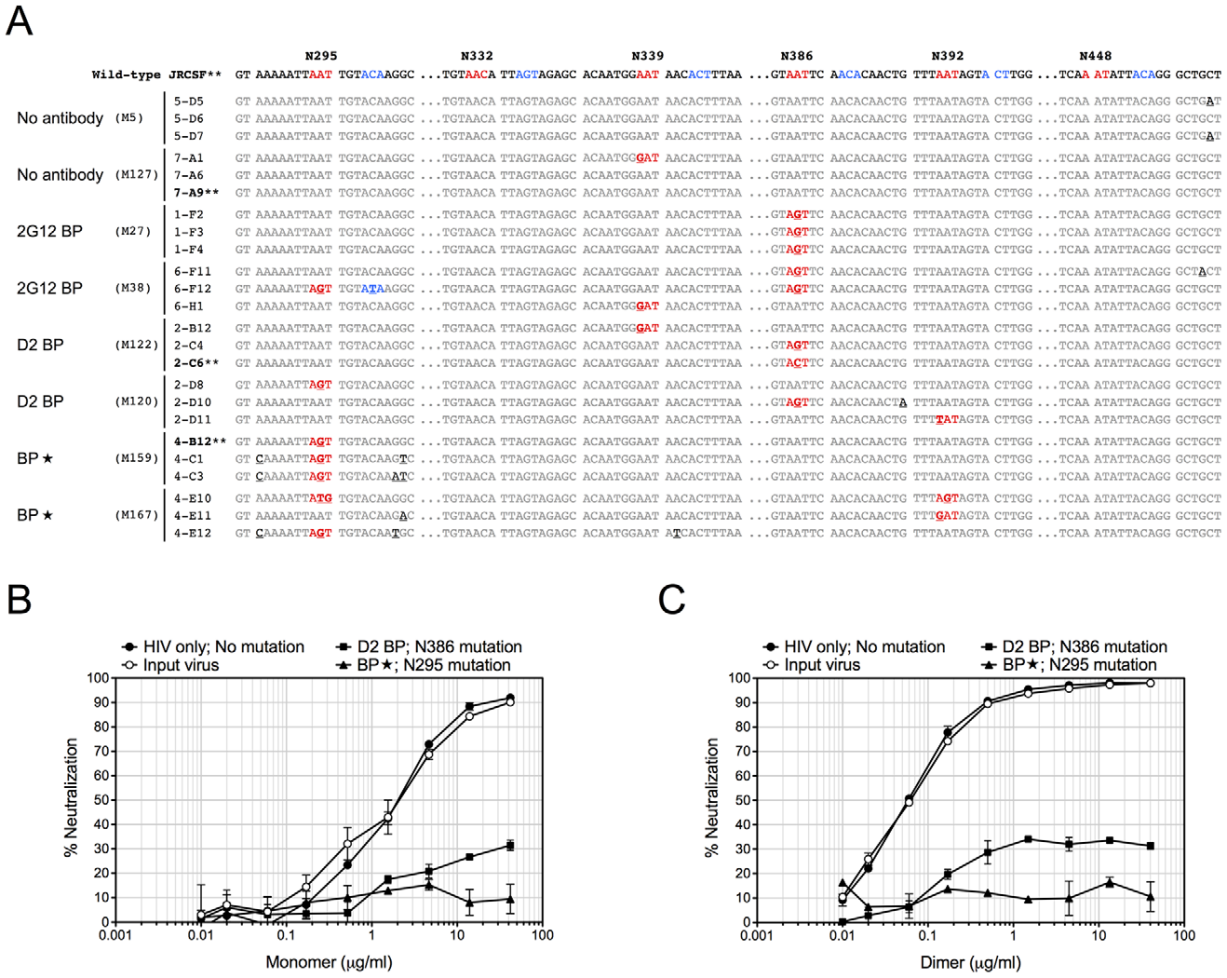
Sequence changes in HIV-1 envelopes and antibody escape. (A) Mouse-derived HIV-1 envelope sequences (regions of interest). Viral RNA was extracted from mouse plasma 4 weeks after HIV-1 challenge, reverse transcribed, and sequenced as described in Materials and Methods. The HIV-1 envelope sequences cloned from mice were aligned to the wild-type JRCSF envelope sequence. The percentages of viral clones with mutations are shown in Table 2. Three sequences from two representative mice of each group are shown in this panel, with the mouse number shown in parentheses. The sequence numbers are also shown next to the sequences. For better viewing, all relevant Asn codons (N295, N332, N339, N386, N392, N448) and their adjacent Ser/Thr codons with mutations are shown in red and blue, respectively, except for the wild-type JRCSF sequence where the wild-type codons are colored. The mutated nucleotides are also bolded and underlined. Some nucleotides other than those that form the carbohydrate anchors were also mutated and they are in black color and underlined. (B) Antibody escape. Four representative envelope genes (labeled with ** in panel A) were subcloned into an expression plasmid for the in vitro neutralization assay. The viral envelope from a D2 BP mouse had a single mutation of N386T. This mutation also occurred in 2G12 BP mice. The viral envelope from a BP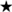 mouse had a mutation of N295S. The viral envelope from an HIV-1-only mouse contained no mutations. The envelope of the virus injected to mice (the input virus) was used as the control. Pseudoviruses were made from these envelopes and in vitro neutralization assay was performed. Neutralization of pseudoviruses by the 2G12 monomer is shown in this panel. (C) Pseudoviruses were made and in vitro neutralization assay was performed as described in panel B. Neutralization of pseudoviruses by the 2G12 dimer is shown in this panel.

Since D2 BP did not completely prevent HIV-1 infection of humanized mice (i.e., HIV-1 viral load was still detectable in the mouse plasma), we asked if increasing the concentration of 2G12 to over 100 µg/ml [11], [12], [13] would provide better protection against HIV-1. Thus, we included a group of mice (named “BP
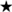
”) that carried large wild-type 2G12 backpacks as a means to maintain both 2G12 monomer and 2G12 dimer at high concentrations in the peripheral blood (Table 1). Our results showed that the large backpacks prevented HIV-1-induced CD4 T cell loss in the peripheral blood (Figure 3A, weeks 1, 2, and 4), suppressed HIV-1 viral load in the mouse plasma (Figure 3B and Figure S2B), decreased the number of p24^+^ cells in the mesenteric lymph node (Figure 3C), and minimized the decrease of CD4 T cell percentage in the spleen (Figure 3D). However, the virus was still detectable in the periphery (Figure 3B). In fact, the overall viral load in BP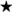 mice was similar to that of D2 BP mice (Figure S2B), suggesting that the concentration of 2G12 dimer required to neutralize HIV-1 in vivo might be as low as 5–25 µg/ml (Table 1, dimer concentrations in the D2 BP group from week 0 to week 4), a level that led to over 70% neutralization of the virus (Figure 3B, comparing D2 BP to the control group lacking antibody). Providing 10-fold more of the 2G12 dimer could potentially prevent CD4 T cell loss in the peripheral blood for a longer period of time (Figure 3A), but it would not prevent HIV-1 entry or further decrease HIV-1 viral load in the plasma (Figure 3B and Figure S2B) or mesenteric lymph node (Figure 3C).

These results showed that a continuous supply of dimeric 2G12 at 5–25 µg/ml during the course of HIV-1 infection is effective at protecting humanized mice against HIV-1 infection.

### Dimeric 2G12 at a low level does not favor HIV-1 envelope mutations at residue N295

Since 2G12 is known to induce HIV-1 escape mutants [16], [19], we extracted viral RNA from the week-4 plasma of 3 or 4 representative mice per experimental group, cloned the JR-CSF envelope gene from viral cDNA, and sequenced at least 10 clones per mouse sample. Some viral clones had spontaneous mutations at residue N339 regardless of the presence of 2G12 and might represent a background in the inoculum (Table 2 and Figure 4A). In addition, both 2G12 BP and D2 BP selected mutations at residue N386. Surprisingly, we observed an unusually high percentage of mutations at residue N295 when the 2G12 concentration was kept at 100 µg/ml or higher (Table 2 and Figure 4A; BP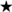). This residue, along with N332 that was not significantly mutated in this study, have been suggested as the key anchors of glycans that form the 2G12 epitope [7]. To assess the sensitivity of mouse-derived viruses to 2G12 neutralization, we performed in vitro neutralization assays using pseudoviruses made with JR-CSF envelope genes that we obtained from mouse plasma samples. Both the input virus (the pseudovirus that shared the same JR-CSF envelope as the inoculum) and the virus with mouse-derived envelope that did not encounter any neutralizing antibody in vivo (HIV-1 only; No mutation) were effectively neutralized by 2G12 monomer and 2G12 dimer in vitro (Figure 4B and 4C); but the half maximal inhibitory concentration (IC_50_) of 2G12 dimer was 33-fold less than the IC_50_ of the monomer, suggesting that the 2G12 dimer was more potent at neutralizing the JR-CSF strain of HIV-1 than the 2G12 monomer. More importantly, we found that the viral envelope from a BP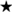 mouse with the mutation N295S caused the pseudovirus to completely escape the neutralization effect of both the 2G12 monomer (Figure 4B) and the 2G12 dimer (Figure 4C). In contrast, a virus variant with a mutation at residue 386 was partially neutralized by the 2G12 monomer and 2G12 dimer. This suggests that, unlike the >100 µg/ml condition (provided by BP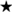), the presence of 2G12 dimer at 5–25 µg/ml (provided by D2 BP) did not select for complete HIV-1 escape mutants.

**Table 2:** Percentage of viral clones mutated at different Asp (N) sites in the JR-CSF envelope gene.

Experimental group	Mouse #	N295	N332	N339	N386	N392	N448	Any N
HIV-1; no antibody	M40	0.0	0.0	14.3	0.0	0.0	0.0	14.3
HIV-1; no antibody	M5	0.0	0.0	7.7	0.0	0.0	0.0	7.7
HIV-1; no antibody	M127	0.0	0.0	31.3	0.0	0.0	0.0	31.3
HIV-1; no antibody	M129	0.0	0.0	11.1	0.0	0.0	0.0	11.1
HIV-1; 2G12 BP	M27	0.0	0.0	0.0	**82.4**	0.0	0.0	82.4
HIV-1; 2G12 BP	M13	0.0	0.0	0.0	**42.9**	0.0	0.0	42.9
HIV-1; 2G12 BP	M15	0.0	0.0	20.0	**12.5**	0.0	0.0	18.2
HIV-1; 2G12 BP	M38	6.7	0.0	6.7	**40.0**	0.0	0.0	20.0
HIV-1; D2 BP	M122	0.0	0.0	17.6	**20.0**	0.0	0.0	35.3
HIV-1; D2 BP	M120	8.3	0.0	8.3	**8.3**	16.7	0.0	41.7
HIV-1; D2 BP	M117	7.1	7.1	0.0	**7.1**	7.1	0.0	28.6
HIV-1; D2 BP	M116	0.0	0.0	0.0	**87.5**	0.0	0.0	87.5
HIV-1; BP 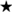	M159	**66.7**	0.0	0.0	0.0	0.0	0.0	66.7
HIV-1; BP 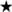	M165	**15.4**	0.0	0.0	0.0	0.0	0.0	15.4
HIV-1; BP 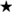	M167	**23.5**	0.0	11.8	11.8	58.8	11.8	82.4

Therefore, our results showed that although constant administration of 2G12 at high concentrations was potent at protecting humanized mice from HIV-1 infection in vivo, it resulted in HIV-1 envelope mutations that could completely escape 2G12 neutralization. However, at least over the time-course of our experiments, a low level of 2G12 dimer did not specifically select the same mutations, providing an additional benefit to its high potency.

## Discussion

In this study, we used a humanized mouse model to investigate the in vivo potency of dimeric 2G12 in controlling HIV-1 infection. This mouse model supports human hematopoietic development, provides human CD4 T cells as natural targets of HIV-1 infection, and allows for possible selection of viral resistance [27]. Using these mice, we first examined the stability and protective effects of monomeric and dimeric forms of 2G12 in HIV-1-challenged humanized mice by passively transferring purified antibodies. We found that the 2G12 dimer had a longer in vivo half-life and was more potent than the 2G12 monomer at controlling HIV-1 infection in vivo. The elimination half-life of the 2G12 dimer was 3.5 days in humanized mice and comparable to the reported elimination half-life (3.2 days) of human IgG1 in mice [28]. This is shorter than the half-life of human IgG1 in humans [18] but correlates with the difference in body weight between mice and humans [29]. To investigate whether a continuous supply of the 2G12 monomer would overcome its poor in vivo efficacy, we next used a backpacking approach to provide the antibody continuously. Using wild-type 2G12 as the backpacked gene, we achieved a sustained level of 2G12 monomer and dimer in the mouse plasma. However, constant delivery of 2G12 monomer plus a small amount of 2G12 dimer at a low level (1–4 µg/ml dimer for the first 3 weeks and 16.6 µg/ml dimer after 4 weeks) did not protect the mice from HIV-1 infection. In contrast, backpacks containing the D2 mutant, which produced increased levels of 2G12 dimer (60% monomer, 40% dimer) provided effective protection against HIV-1 by maintaining a 2G12 dimer concentration of 5–25 µg/ml in the mouse plasma. Thus, our results suggest that, administered either through a single injection or continuously, dimeric 2G12 is a more potent prophylactic anti-HIV-1 antibody than 2G12 monomer.

Several in vivo studies have estimated that concentrations of 2G12 of 100 µg/ml or higher exert a protective effect against HIV-1 when the virus is given at a 50% tissue culture infective dose (TCID50) of 500—5,000 [11], [12], [13]. In order to establish a robust and consistent infection in humanized mice, we administered HIV-1 intravenously at a dosage of 400 ng p24, or a TCID50 of 400,000. Although sterilizing immunity was not achieved in this study, we found that, even with high-dose HIV-1 challenge, 2G12 monomer and dimer at combined concentrations of 100 µg/ml or higher could significantly reduce the severity of HIV-1 infection in the humanized mice (Figure 3). More importantly, the D2 BP that delivered 2G12 at a much lower concentration exerted a similar protective effect against HIV-1. In particular, D2 BP provided the 2G12 dimer at 5–25 µg/ml, which was sufficient to prevent peripheral blood CD4 T cell loss (Figure 3A) and suppress the increase of the viral load following HIV-1 infection (Figure 3B and Figure S2B). Therefore, 2G12 dimer represents a promising prophylactic reagent against HIV-1 in vivo because it neutralizes HIV-1 at a relatively low concentration.

Having a low effective concentration is not the only advantage of the 2G12 dimer as a protective reagent against HIV-1. 2G12 is known to select HIV-1 escape mutants both in vitro [30], [31] and in vivo [16], [30], [31], with in vivo escape mutants detectable as early as 4 weeks after HIV-1 inoculation [16], [30], [31]. Here we analyzed the diversity of HIV-1 viral RNA isolated from the mouse plasma, focusing on regions of the JR-CSF envelope gene where 2G12 epitope-containing carbohydrates would attach [7], [32]. We found that while low levels of 2G12 dimer induced mutations at residue N386, 2G12 at monomer plus dimer concentrations of >100 µg/ml specifically selected mutations at another residue (Table 2). This residue, N295, has been suggested to be one of the two central players in the interaction between 2G12 and its carbohydrate epitope [7]. A mutation at N295 would be more likely to allow HIV-1 to escape 2G12 neutralization than mutations at other sites such as N386 (Figure 4B and Figure 4C). Thus, at least over the time-course of our experiments, dimeric 2G12 provided protection against HIV-1 without selecting for complete HIV-1 escape mutants.

In summary, we found in the present study that dimeric 2G12, or the D2 mutant that increases the production of dimeric 2G12, might be potential prophylactic reagents against HIV-1. However, more research is necessary to characterize the tissue distribution of dimeric 2G12 and its in vivo antibody-dependent cellular cytotoxicity activity. It is also important to assess the immunogenicity of 2G12 in its dimeric form since it is twice the size of a typical IgG. In addition, the pharmacokinetics of dimeric 2G12 should be carefully established in human studies, as the half-life of the antibody in humans is likely to be different from that in humanized mice. Furthermore, because the neutralization spectrum of 2G12 is not particular good when tested against a large panel of HIV-1 isolates [10] and neutralizing antibodies have demonstrated synergy when combined together [33], the 2G12 dimer may be more beneficial when used as part of an antibody cocktail to protect people from HIV-1 infection.

## Materials and Methods

### Ethics statement

This study was carried out in strict accordance with the recommendations in the Guide for the Care and Use of Laboratory Animals of the National Institutes of Health. The protocol was approved by the Institutional Animal Care and Use Committee (IACUC) of California Institute of Technology (Animal Assurance Number: A3426-01). All animal experiments were conducted under IACUC protocols 1536-09G and 1547-08G.

### Expression and purification of 2G12

The wild-type 2G12 heavy chain gene (IgG1) and a Myc-tagged 2G12 light chain gene were linked by an F2A sequence and subcloned into a lentivector. The vector is a third-generation, self-inactivating lentiviral vector backbone based on pHRST [34], [35]. Briefly, the StuI fragment of pHRST containing a complete viral genome was ligated into the pUC19 backbone to remove exogenous flanking genomic sequences. PCR-cloning was employed to introduce restriction sites flanking the promoter and transgene to facilitate subsequent cloning. Further modifications were made to pHAGE6 to remove extraneous viral sequences with no effect on virus function (A.B., to be published elsewhere). Lentiviruses were then generated by transient transfection of HEK-293T cells using the Trans-IT reagent (Mirus Bio; Madison, WI) and used to create a 293T stable cell line that produced 2G12. The 2G12-expressing, adherent stable cell line was adapted for growth in suspension for large-scale production of 2G12 at the Caltech Protein Expression Center. Cell culture supernatants were collected and passed over protein A resin (Pierce Biotechnology; Rockford, IL), and eluted using using pH 3.0 citrate buffer. Protein A eluates were immediately neutralized and then subjected to size exclusion chromatography in 20 mM Tris pH 8.0, 150 mM NaCl using a Superdex 200 16/60 (GE Healthcare). Fractions corresponding to monomer and dimer were collected and then separately passaged over a Superdex 200 10/30 column (GE Healthcare) to remove contaminating amounts of monomer or dimer from the separated purified species.

### Humanized mice, passive transfer, and HIV-1 challenge

Frozen human cord blood CD34^+^ cells from single donors were purchased from AllCells (Emeryville, CA) or Lonza (Basel, Switzerland). One-day-old Rag2^−/−^γ_c_^−/−^ pups were irradiated and intrahepatically (i.h.) injected with 0.1-0.2×10^6^ human cord blood CD34^+^ cells per pup. Mice were then screened for human CD45^+^ cells at 6 weeks of age and those with good reconstitution were chosen for the study (Figure S1A). For passive transfer experiments, one single dose of 0.5 mg/mouse of purified 2G12 dimer or 2G12 monomer was injected retro-orbitally (i.v.) into 4-month-old humanized mice 1 day before HIV-1 challenge. The HIV-1 JR-CSF plasmid was obtained from NIH AIDS Research and Reference Reagent Program and transiently transfected into 293T cells to produce infectious HIV-1 particles. The culture medium containing HIV-1 was then harvested and titered using the p24 ELISA kit from PerkinElmer (Waltham, MA). The virus was injected (i.v.) at 400 ng p24/mouse. For non-HIV-1 mice, conditioned medium was injected as the control. All mice involved in this study were age-matched since the CD4:CD8 ratio naturally increased with the age of these mice.

### Generation of backpacked mice

Wild-type 2G12 and D2 mutant genes were cloned into lentiviral vectors. Lentiviruses were then generated and used to create stable cell lines that produced wild-type 2G12 and D2, respectively. The parent cell line was a stable 293T cell line that expressed herpes simplex virus thymidine kinase (TK), so the progeny lines were named 293T/TK/2G12 and 293T/TK/D2 cell lines. When well-reconstituted humanized mice were 3-month-old, 1×10^6^ of backpacked cells were injected (s.c.) on the back of the mice at the lower right side. Backpack size (length × width) was measured weekly and controlled by injection (i.p.) of 62.5 µg or 125 µg (depending on the backpack size) of ganciclovir (Sigma; St. Louis, MO) per mouse after HIV-1 challenge and when the backpack size reached 1.5 cm^2^.

### Sample collection

Weekly blood samples were obtained retro-orbitally and the plasma was immediately separated from blood cells and stored for viral RNA extraction and Myc-specific ELISA (see below for details). The peripheral blood mononuclear cells after antibody staining were analyzed by the FACSCalibur (BD Biosciences; San Jose, CA). Mice were sacrificed 4 weeks after HIV-1 challenge. Blood, spleen, thymus, and mesenteric lymph node were collected for flow cytometry analysis or fixation in formalin. The fixed tissues were then send to University of California, Los Angeles for immunohistochemical analysis.

### Determination of 2G12 concentrations

Mouse plasma was diluted 1∶10, 1∶100, and 1∶1000 in sample diluent and heat-inactivated at 55°C for 1 h. Myc-tagged 2G12 was captured by anti-human IgG-Fc (Bethyl Laboratories; Montgomery, TX) and detected by anti-Myc conjugated with horseradish peroxidase (Bethyl Laboratories; Montgomery, TX). The plates were read at 450 nm on a SpectroMax Reader (Molecular Devices, Sunnyvale, CA) after the addition of the TMB substrate and the stop solution. In passive transfer experiments, the half-life of the elimination phase (β phase), which took place after the redistribution phase, was determined using a one-phase exponential decay model using data points from week 0 (24 h after the injection of 2G12 monomer or dimer) to week 4. The half-lives were estimated as 3.5±0.9 days for the 2G12 dimer and 0.9±0.2 days for the 2G12 monomer. In backpacking experiments where both 2G12 monomer and dimer were present in the plasma, we determined their individual concentrations by calculating the monomer:dimer ratios as following: 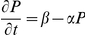
where *P* =  protein (monomer or dimer), *β* =  production rate; *α* =  degradation rate. Assuming that at the time of HIV-1 challenge (4 weeks after backpack injection), the monomer and dimer had reached their individual steady state (i.e. 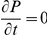),



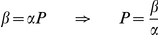



If the dimer had a production rate of *β* and a degradation rate of *α*, then the monomer should have a production rate of 3.5*β* (78% monomer versus 22% dimer produced from 2G12 backpacks) and a degradation rate of 3.9*α* (dimer:monomer ratio of half-lives 3.5/0.9 = 3.9) for 2G12 backpacks. Thus, 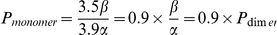


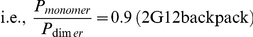



Therefore, the 2G12 monomer and dimer concentrations were calculated as:
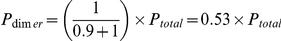


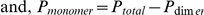
where *P_total_* =  total 2G12 concentration as measured by Myc-specific ELISA. For D2 backpacks, since the dimer's production rate was 1.5*β* (60% monomer versus 40% dimer produced from D2 backpacks) and the degradation rate stayed the same,
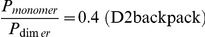


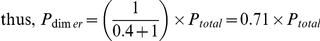



### In-house HIV-1 viral load assay

Viral RNA was extracted from mouse plasma using QIAamp Viral RNA Mini Kit from Qiagen (Valencia, CA). The RNA (200 ng) was reverse transcribed and quantified using the Taqman RNA-to-CT One-Step Kit (Applied Biosystems; Foster City, CA) and the Eppendorf Realplex real-time PCR system (Hauppauge, NY). The primers were designed to anneal to the pol region of the HIV-1 genome within the first intron, so that only unspliced viral RNA could be detected. The primer sequences were: forward primer, 5′-CAA TGG CAG CAA TTT CAC CA-3′; reversed primer, 5′-GAA TGC CAA ATT CCT GCT TGA-3′. The probe sequence was: 5′-/56-FAM/CCC ACC AAC AGG CGG CCT TAA CTG/36-TAMSp/-3′. HIV-1 RNA standard was generated using the Riboprobe T7/SP6 kit from Promega (Madison, WI) and the pGEM FL2 plasmid was provided by Dr. Dong Sung An at University of California, Los Angeles. The detection limit of the assay was 20,000 HIV-1 copies/ml mouse plasma.

### Sequencing analysis

Viral RNA was extracted from mouse plasma using QIAamp Viral RNA Mini Kit from Qiagen (Valencia, CA). The RNA (500 ng) was reverse transcribed and amplified using the SuperScript III One-Step RT-PCR System with Platinum Taq High Fidelity from Invitrogen (Carlsbad, CA). The primer sequences were: JR-CSF env forward primer, 5′-GGC AAT GAG AGT GAA GGG GAT CAG-3′; JR-CSF env reversed primer, 5′-CAT CTT ATA GCA AAG CCC TTT CCA AGC C-3′. The primers flanked the whole 2.5-kb envelope gene. The PCR product was then gel-purified and cloned into the TOPO vector using the TOPO XL PCR Cloning Kit from Invitrogen (Carlsbad, CA). More than 10 clones were picked for each RNA sample. The plasmids were then extracted and sent to sequencing at Laragen (Los Angeles, CA) or Sequetech (Mountain View, CA). The sequencing primer was 5′-GTC AGC ACA GTA CAA TGT ACA CAT GGA ATT AG -3′ and annealed upstream of the Asn residues that linked 2G12 epitope-containing carbohydrate chains [7]. Mutations at N295, N332, N339, N386, N392, N448 and adjacent Ser/Thr residues were then analyzed.

### In vitro neutralization assay

We used a previously described pseudovirus neutralization assay, which measures the reduction in luciferase reporter gene expression in the presence of 2G12 monomer or dimer following a single round of pseudovirus infection in TZM-bl cells [20]. Pseudoviruses were generated by cotransfection of 293T cells with an envelope expression plasmid and a replication-defective backbone plasmid. (For envelope expression, viral RNA was extracted from mouse plasma 4 weeks after HIV-1 challenge and reverse transcribed. The complete envelope gene was amplified from viral cDNA and the PCR product was then gel-purified and cloned into the pcDNA3 vector.) Each 2G12 protein was tested in triplicate with a 3-fold dilution series, and incubated with the pseudoviruses (250 infectious viral units per well) for 1 h at 37°C. After the incubation, 10,000 TZM-bl cells were added to each well, followed by incubation for 2 days. Cells were then lysed and assayed for luciferase expression by using Bright-Glo (Promega; Madison, WI) and a Victor3 luminometer (Perkin-Elmer; Waltham, MA).

## Supporting Information

Figure S1 Rag2^−/−^γ_c_^−/−^ mice were intrahepatically (i.h.) injected with 0.1∼0.2×10^6^ human CD34^+^ hematopoietic stem and progenitor cells at 1 day of age to become humanized mice. (A) Mice were screened for the percentages of human CD45^+^ cells in the peripheral blood at 6 weeks of age and those with good reconstitution (>20% CD45^+^ cells) were chosen for the study. The reconstitution rates were not different among the groups. (B) Humanized mice were injected intravenously (i.v.) with 0.5 mg of purified 2G12 monomer (n = 6) or 2G12 dimer (n = 5) at 4 months of age and challenged by the JR-CSF strain of HIV-1 (i.v.; 400 ng of p24) one day after the passive transfer. After the mice were sacrificed, mesenteric lymph nodes (mLN) were harvested, fixed, and sectioned for immunohistochemical analysis of CD4 and CD8 expression. The numbers of CD4^+^ and CD8^+^ cells were counted manually and the ratios of CD4:CD8 are shown. (C) After the mice were sacrificed, CD4 and CD8 T cell populations in the thymus were measured by flow cytometry. The percentages of CD4^+^CD8^−^ cells in CD45^+^ human thymocytes were plotted.
(0.44 MB TIF)

Figure S2Rag2^−/−^γ_c_^−/−^ mice were intrahepatically (i.h.) injected with 0.1∼0.2×10^6^ human CD34^+^ hematopoietic stem and progenitor cells at 1 day of age. When the mice were 3-month-old, we delivered 2G12 through subcutaneous (s.c.) injection of a cell line on the back of the mice. The cell line, 293T/TK/2G12, formed controllable backpacks on the mice (see the text and Materials and Methods for details). The backpack size was closely monitored biweekly and the prodrug ganciclovir was injected (i.p.) after HIV challenge and when the backpacks exceeded the size limit of 1.5 cm^2^. The concentrations of 2G12 (monomer plus dimer) produced in the blood were monitored by ELISA. (A) Analysis of the backpack size versus the plasma level of 2G12 showed significant correlation (R^2^ = 0.53, *p*<0.0001) between the two. Seventy-one data points from weeks 2–7 after 293T/TK/2G12 injection were plotted on the graph. Earlier data points were excluded because the backpacks were not detectable at the time. (B) The backpacks expressing wild-type 2G12 were named 2G12 backpacks (“2G12 BP”; n = 8) whereas the ones expressing the D2 mutant were named D2 backpacks (“D2 BP”; n = 7). Another group of mice were made to carry wild-type 2G12-expressing backpacks (“BP
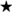
”; n = 7) till the plasma concentrations of 2G12 (monomer plus dimer) reached 100 µg/ml before HIV inoculation. Viral RNA was extracted from mouse plasma after HIV infection and the viral load was measured. Area under the curve (AUC) of the 4 groups from week 0 to week 4 was calculated and plotted. Both “D2 BP” and “BP
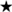
” groups had significantly lower viral load than the “HIV; No Ab” control (*p*<0.01). (C) After the mice were sacrificed, the spleens were harvested, fixed, and sectioned for immunohistochemical analysis of HIV-1 p24. The numbers of p24^+^ cells were counted manually and presented as the number of cells per mm^2^ area of the specimen.(0.37 MB TIF)
