# Supplementary figures and images for: Dimeric 2G12 as a Potent Protection against HIV-1
Source: PLoS Pathog. 2010 Dec 16;6(12):e1001225. doi: 10.1371/journal.ppat.1001225 (PMC3002980; doi:10.1371/journal.ppat.1001225)

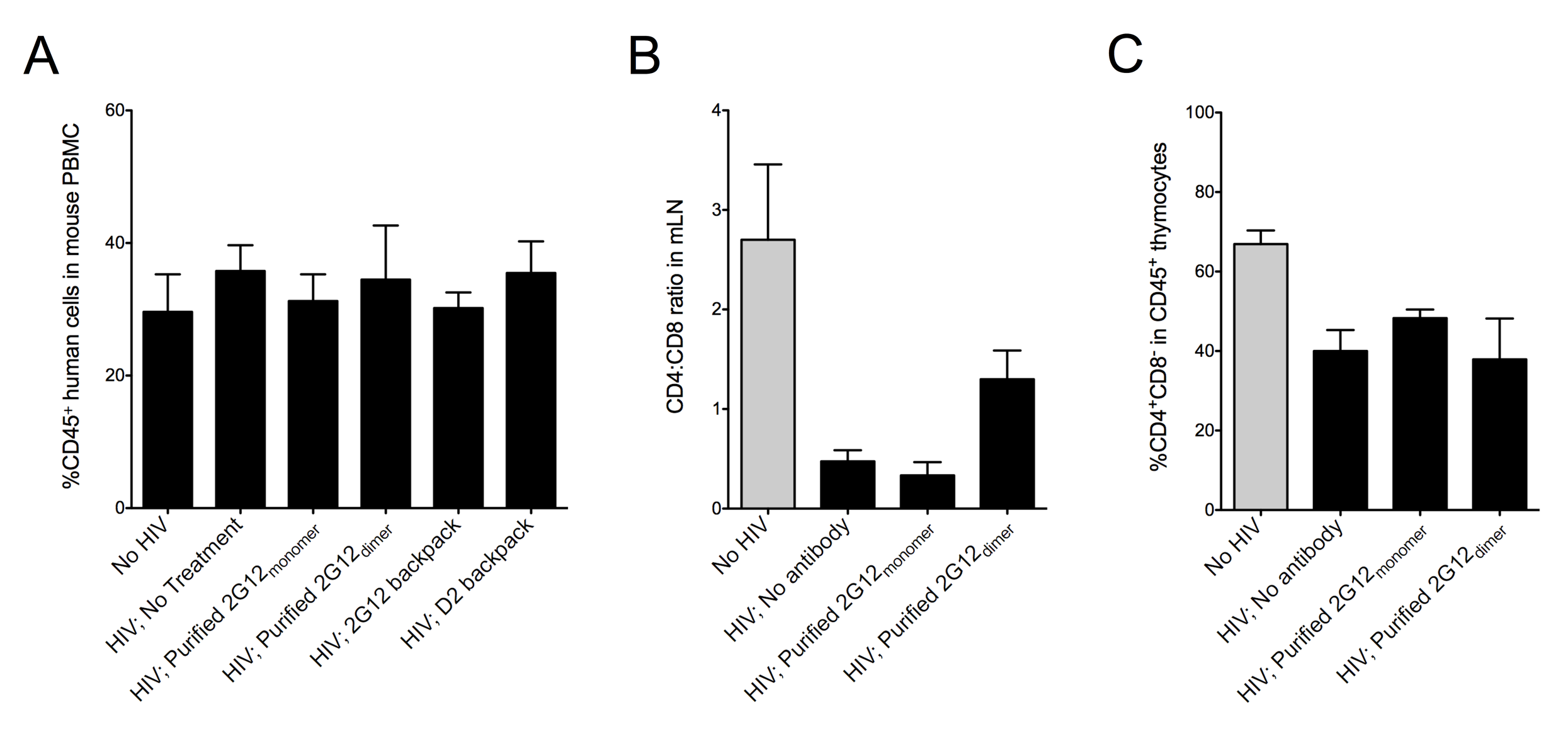

Supplement: Figure S1 — Rag2−/−γc −/− mice were intrahepatically (i.h.) injected with 0.1∼0.2×10^6^ human CD34+ hematopoietic stem and progenitor cells at 1 day of age to become humanized mice. (A) Mice were screened for the percentages of human CD45+ cells in the peripheral blood at 6 weeks of age and those with good reconstitution (>20% CD45+ cells) were chosen for the study. The reconstitution rates were not different among the groups. (B) Humanized mice were injected intravenously (i.v.) with 0.5 mg of purified 2G12 monomer (n = 6) or 2G12 dimer (n = 5) at 4 months of age and challenged by the JR-CSF strain of HIV-1 (i.v.; 400 ng of p24) one day after the passive transfer. After the mice were sacrificed, mesenteric lymph nodes (mLN) were harvested, fixed, and sectioned for immunohistochemical analysis of CD4 and CD8 expression. The numbers of CD4+ and CD8+ cells were counted manually and the ratios of CD4:CD8 are shown. (C) After the mice were sacrificed, CD4 and CD8 T cell populations in the thymus were measured by flow cytometry. The percentages of CD4+CD8− cells in CD45+ human thymocytes were plotted. (0.44 MB TIF) [file ppat.1001225.s001.tif]

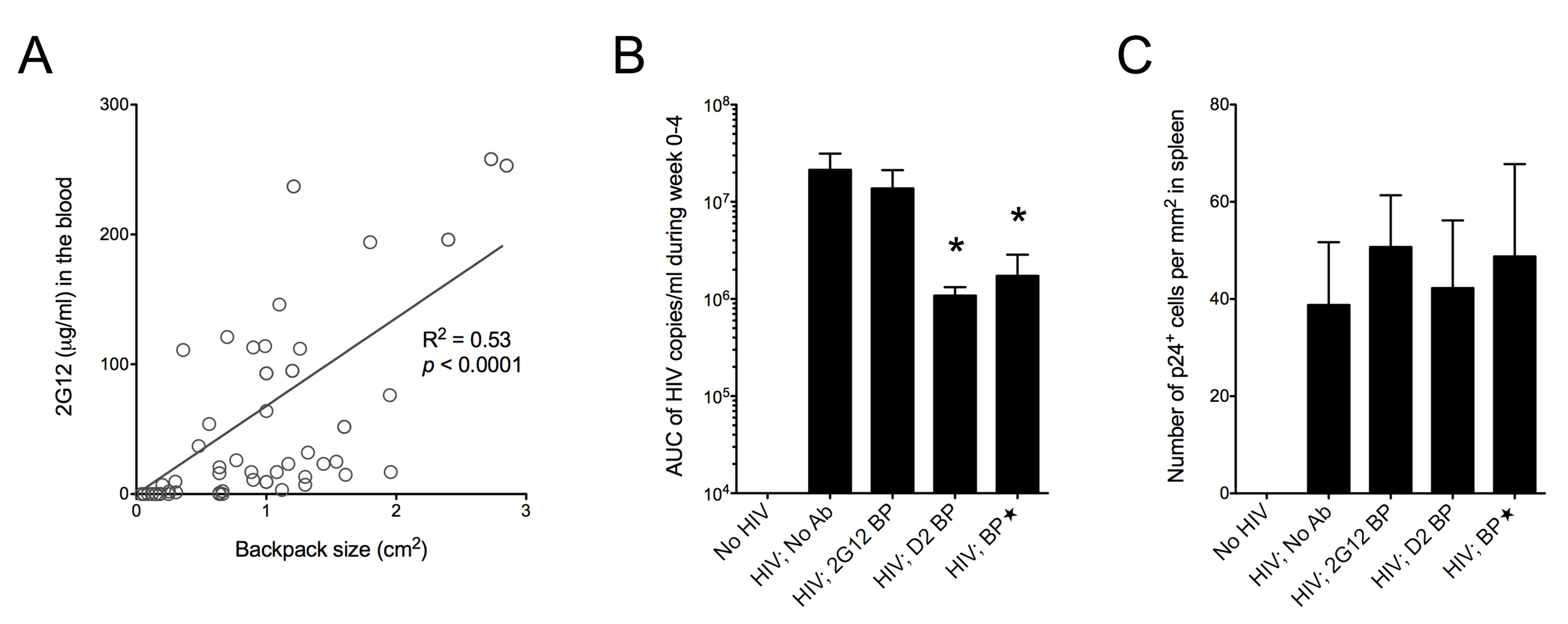

Supplement: Figure S2 — Rag2−/−γc −/− mice were intrahepatically (i.h.) injected with 0.1∼0.2×10^6^ human CD34+ hematopoietic stem and progenitor cells at 1 day of age. When the mice were 3-month-old, we delivered 2G12 through subcutaneous (s.c.) injection of a cell line on the back of the mice. The cell line, 293T/TK/2G12, formed controllable backpacks on the mice (see the text and Materials and Methods for details). The backpack size was closely monitored biweekly and the prodrug ganciclovir was injected (i.p.) after HIV challenge and when the backpacks exceeded the size limit of 1.5 cm2. The concentrations of 2G12 (monomer plus dimer) produced in the blood were monitored by ELISA. (A) Analysis of the backpack size versus the plasma level of 2G12 showed significant correlation (R2 = 0.53, p<0.0001) between the two. Seventy-one data points from weeks 2–7 after 293T/TK/2G12 injection were plotted on the graph. Earlier data points were excluded because the backpacks were not detectable at the time. (B) The backpacks expressing wild-type 2G12 were named 2G12 backpacks (“2G12 BP”; n = 8) whereas the ones expressing the D2 mutant were named D2 backpacks (“D2 BP”; n = 7). Another group of mice were made to carry wild-type 2G12-expressing backpacks (“BP”; n = 7) till the plasma concentrations of 2G12 (monomer plus dimer) reached 100 µg/ml before HIV inoculation. Viral RNA was extracted from mouse plasma after HIV infection and the viral load was measured. Area under the curve (AUC) of the 4 groups from week 0 to week 4 was calculated and plotted. Both “D2 BP” and “BP” groups had significantly lower viral load than the “HIV; No Ab” control (p<0.01). (C) After the mice were sacrificed, the spleens were harvested, fixed, and sectioned for immunohistochemical analysis of HIV-1 p24. The numbers of p24+ cells were counted manually and presented as the number of cells per mm2 area of the specimen. (0.37 MB TIF) [file ppat.1001225.s002.tif]
